# One-Tenth of the Top 50 Pediatric Orthopedic Hospitals Provide Compliant Price Transparency Information for 15 Common Pediatric Orthopedic Procedures

**DOI:** 10.7759/cureus.80355

**Published:** 2025-03-10

**Authors:** Nicholas G Belt, Austin Lee, Andrew Moyal, Robert Burkhart, Victoria Nedder, Raymond Liu

**Affiliations:** 1 Medical School, Case Western Reserve University School of Medicine, Cleveland, USA; 2 Department of Orthopaedic Surgery, University Hospitals Cleveland Medical Center, Cleveland, USA; 3 Department of Orthopaedic Surgery, Case Western Reserve University School of Medicine, Cleveland, USA

**Keywords:** cms compliance, healthcare costs, hospital price variability, pediatric orthopedics, price transparency

## Abstract

Introduction

In 2019, Centers for Medicare and Medicaid Services (CMS) mandated hospitals to provide publicly available chargemasters to aid in transparency of pricing for hospital procedures. Despite the mandate, many orthopedic hospitals do not comply with CMS guidelines. The goals of this study are to (1) assess the compliance of the top 50 U.S. children’s orthopedic hospitals with the CMS mandate and (2) analyze variation in pricing for common orthopedic procedures among these hospitals.

Methods

The top 50 pediatric orthopedic hospitals within the United States were selected based on U.S. News and World Report. Fifteen common pediatric orthopedic procedures were then selected based on literature and internal institutional volume. The website of each hospital was searched for the required downloadable chargemaster and/or a user-friendly online tool to provide pricing for each procedure. Compliance was assessed by the ability to find payer-negotiated charges, gross charges, and cash-based cost for each procedure. Hospitals were deemed compliant if they met all guidelines, pseudo-compliant if they met any of the above guidelines, and noncompliant if they met no guidelines.

Results

Only 10% (five of 50) of the hospitals complied with all 15 procedures, while an additional 32% (16 of 50) were pseudo-compliant for at least one of the specific procedures searched. A total of seven hospitals listed cash prices, 10 hospitals listed gross charges, and 12 hospitals listed payer-negotiated charges. The widest range for gross charge was Current Procedural Terminology (CPT) code 23462 (arthroscopic Bankart repair), ranging from $3,012 to $109,320. The range of charges dramatically differed from the gross price for all procedures.

Conclusions

Only 10% of the top 50 pediatric orthopedic hospitals in the United States are compliant with the CMS mandate for price transparency. Furthermore, the cost of each procedure varied widely depending on hospital and type of price reported (gross, cash, and payer-negotiated). These substantial shortcomings call for an evaluation of the current strategies being employed to improve price transparency in healthcare.

## Introduction

Healthcare price transparency requires disclosure of information on the cost of healthcare [1]. Centers for Medicare and Medicaid Services (CMS) mandated in 2019 that hospital chargemasters be publicly available in a machine-readable file [2]. Price transparency can improve patient knowledge about the cost of care and increase patient involvement in making cost-conscious choices [1-4]. There is also significant variation in prices for care, and price transparency can increase competition in the healthcare system, creating an incentive to lower prices and reduce costs [1,3,4]. Price transparency is especially important in the United States because out-of-pocket costs are common for medical services and depend on factors such as the patient’s coverage status, type of insurance, and whether the service is in- or out-of-network [5]. Additionally, many individuals have inadequate health literacy and low confidence in their ability to use insurance to access healthcare [6,7].

Despite the 2019 CMS mandate, few institutions are currently compliant with price transparency regulations [2,4,8-12]. One study found that 51.5% of a sample of 5,288 U.S. hospitals were not compliant with the mandate [2]. Most healthcare price websites only report billed charges (rather than out-of-pocket charges), which a patient is rarely fully responsible for [8,9]. Many websites also tend to report only facility fees and not professional fees, underestimating the total price [8,9]. Despite attempted enforcement by the CMS mandate, estimating the price of care continues to pose a large challenge to both patients and providers alike.

Some pediatric literature has attempted to analyze price transparency for individual services, such as imaging, tonsillectomy, ACL reconstructions, and radius fractures. While data shows CMS compliance rates from 20% to 44% and high variability in pricing, these studies relied on phone-call scripts rather than use of the required online chargemasters [9-12]. Given these limitations, further research is required to better understand pediatric price transparency, especially within pediatric orthopedics. Therefore, we asked the following: (1) What is the compliance rate for a variety of common pediatric procedures among the top 50 U.S. children’s orthopedic hospitals? and (2) How do prices compare among these hospitals?

## Materials and methods

Study design, hospital selection, and procedure selection

The top 50 hospitals for pediatric orthopedic surgery were selected for use in this study, as ranked by U.S. News and World Report [13]. Basic data for each hospital was collected, including size by number of beds, U.S. census region, type of ownership, and for-profit vs. not-for-profit. Because our study utilized publicly available data, this study was exempt from institutional review board approval.

Fifteen common pediatric orthopedic procedures were selected, informed by a combination of contemporary literature and internal institutional volume [14,15]. The procedures selected were: 22802 (adolescent idiopathic scoliosis [AIS]: fusion 7-12 levels), 22843 (AIS: instrumentation 7-12 levels), 23462 (arthroscopic Bankart repair), 24538 (supracondylar humerus fracture: closed reduction percutaneous pinning [CRPP]), 24545 (supracondylar humerus fracture: open reduction internal fixation [ORIF]), 25565 (both bone forearm fracture closed reduction), 25574 (both bone forearm fracture ORIF), 27176 (surgical pinning of slipped capital femoral epiphysis), 27502 (midshaft femur fracture, closed treatment of femoral shaft fracture, with manipulation with or without skin or skeletal traction), 27506 (midshaft femur fracture open fixation with intramedullary device), 29860 (diagnostic hip arthroscopy), 29880 (arthroscopic partial meniscectomy), 29888 (arthroscopic anterior crucial ligament reconstruction), 29914 (femoroplasty), and 29916 (arthroscopic labral repair of the hip).

Under the CMS price transparency rule, every hospital is mandated to publish a comprehensive list of standard charges for all the services and items they offer [16]. This list must be accessible online in a downloadable, machine-readable format. The required information for each service or item includes gross charges, payer-specific negotiated charges, de-identified minimum and maximum negotiated charges, and discounted cash prices (CMS required prices). The gross charge is the total amount billed by the hospital for services before any discounts or insurance adjustments are applied [16]. Payer-specific negotiated charge is the charge that a hospital negotiated with a third-party payer for an item or service [16]. The de-identified minimum and maximum negotiated charges are the lowest and highest charges that a hospital negotiated with all third-party payers [16]. The discounted cash price is the charge that applies to an individual who pays cash, or cash equivalent, for an item or service [16]. Importantly, this information must be made available to patients free of charge, and access should not require any personal identifying information.

What is the compliance for a variety of common pediatric procedures among the top 50 U.S. children’s orthopedic hospitals?

Comprehensive search of hospital chargemasters was completed between December 1 and December 15, 2023, by three authors (initials blinded for peer review). All Current Procedural Terminology (CPT) codes were analyzed using machine-downloadable files or user-friendly price-estimator tools. If accessing pricing information necessitated personal insurance status, the reviewers selected options associated with being uninsured. In instances where personal information was required for access, the generic name of “John Doe” with a birthdate of January 1, 1980, was utilized. If use of the above tools did not provide pricing for specific CPT codes, a second search was completed utilizing standardized keywords such as “forearm fracture,” “humerus fracture,” “arthroplasty,” “knee,” “hip,” or “femur.”

The CMS required prices were collected for each procedure. Hospitals providing all five datapoints were deemed compliant, those offering one to four datapoints were considered pseudo-compliant, and those offering no data were deemed noncompliant. In the process of searching for the above charges, the following factors were analyzed to assess consumer usability: (1) possible language options, (2) the time required to locate procedure prices, (3) availability of contact information for inquiries, and (4) the provision of procedure descriptions in common terms.

How do prices compare among the top 50 U.S. children’s orthopedic hospitals?

For all CPT codes listed, data was collected pertaining to the CMS required prices. Charges were organized and compared based on procedure type. A specific note was made of the average, maximum, and minimum charges. No inferential statistical analysis was completed.

## Results

Characteristics of included hospitals

All 50 of the hospitals included in the study were non-profit teaching hospitals located in Urban areas (Table 6, Appendix). When stratified by U.S. census regions, 21 (42%) hospitals were in the South, followed by 11 (22%) in the Midwest (11 of 50), 11 (22%) in the West, and seven (14%) in the Northeast. Most hospitals were private (76%). The bed size ranged from 125 to 973, with most hospitals falling between 200 and 400 beds (Table 1). 

**Table 1:** Hospital characteristics by size, U.S. census region, and type of ownership

Characteristic	Number of hospitals
Hospital size	
<200 beds	14
200-400 beds	25
>400 beds	11
U.S. census region	
Midwest	11
Northeast	7
South	21
West	11
Hospital ownership	
Public	12
Private	38

What is the compliance for a variety of common pediatric procedures among the top 50 U.S. children’s orthopedic hospitals?

Only five (10%) of the hospitals were compliant with all of the requirements stated in the Hospital Price Transparency final rule for all 15 procedures [2]. Additionally, 16 (32%) hospitals were pseudo-compliant with at least one of the procedures included (Table 2). When evaluating compliance per CPT code, 14 (28%) and 13 (26%) of hospitals qualified as pseudo-compliant for CPT codes 24538 (supracondylar humerus fracture: CRPP) and 29880 (arthroscopic partial meniscectomy), respectively, representing the two most compliant codes.

**Table 2:** Level of compliance per hospital Compliant (C), pseudo-compliant (P), and noncompliant (N)

Hospital name	Location	CPT 24538	CPT 24545	CPT 27506	CPT 27502	CPT 29888	CPT 29880	CPT 22802	CPT 22843	CPT 25574	CPT 25565	CPT 23462	CPT 29860	CPT 29914	CPT 29916	CPT 27176
Children's Hospital of Philadelphia	Philadelphia, PA	N	N	N	N	N	N	N	N	N	N	N	N	N	N	N
Children's Hospital Los Angeles	Los Angeles, CA	N	N	N	N	N	N	N	N	N	N	N	N	N	N	N
Children's National Hospital	Washington, DC	C	C	C	C	C	C	C	C	C	C	C	C	C	C	C
Texas Children's Hospital	Houston, TX	N	N	N	N	N	N	N	N	N	N	N	N	N	N	N
Johns Hopkins Children's Center	Baltimore, MD	N	N	N	N	N	N	N	N	N	N	N	N	N	N	N
Children's Healthcare of Atlanta	Atlanta, GA	N	N	N	N	N	N	N	N	N	N	N	N	N	N	N
Nationwide Children's Hospital	Columbus, OH	C	C	C	C	C	C	C	C	C	C	C	C	C	C	C
UH Rainbow Babies and Children's Hospital	Cleveland, OH	N	N	N	N	N	N	N	N	N	N	N	N	N	N	N
St. Louis Children's Hospital-Washington University	St. Louis, MO	N	N	N	N	N	N	N	N	N	N	N	N	N	N	N
UPMC Children's Hospital of Pittsburgh-Shriners Hospitals for Children Erie	Pittsburgh, PA	N	N	N	N	N	N	N	N	N	N	N	N	N	N	N
Children’s Hospital Colorado	Aurora, CO	N	N	N	N	N	N	N	N	N	N	N	N	N	N	N
Mayo Clinic Children's Center	Rochester, MN	P	P	P	P	P	P	P	N	P	P	P	P	P	P	P
Lerner Children's Pavilion-Hospital for Special Surgery	New York, NY	N	N	N	N	N	N	N	N	N	N	N	N	N	N	N
Children's Mercy Kansas City	Kansas City, MO	N	N	N	N	N	N	N	N	N	N	N	N	N	N	N
Levine Children's Hospital	Charlotte, NC	N	N	N	N	N	N	N	N	N	N	N	N	N	N	N
UC Davis Children's Hospital-Shriners Children's Northern California	Sacramento, CA	N	N	N	N	N	N	N	N	N	N	N	N	N	N	N
North Carolina Children's Hospital at UNC	Chapel Hill, NC	N	N	N	N	N	N	N	N	N	N	N	N	N	N	N
Monroe Carell Jr. Children’s Hospital at Vanderbilt	Nashville, TN	P	P	P	P	N	P	P	P	P	P	N	P	P	P	P
Duke Children's Hospital and Health Center	Durham, NC	P	N	N	N	N	N	N	N	N	N	N	N	N	N	N
Lucile Packard Children's Hospital Stanford	Palo Alto, CA	N	N	N	N	N	N	N	N	N	N	N	N	N	N	N
Valley Children's Healthcare and Hospital	Madera, CA	N	N	N	N	N	N	N	N	N	N	N	N	N	N	N
Dayton Children's Hospital	Dayton, OH	C	C	C	C	C	C	C	C	C	C	C	C	C	C	C
NewYork-Presbyterian Children's Hospital-Columbia and Cornell	New York, NY	N	N	N	N	N	N	N	N	N	N	N	N	N	N	N
University of Iowa Stead Family Children's Hospital	Iowa City, IA	N	N	N	N	N	N	N	N	N	N	N	N	N	N	N
Cleveland Clinic Children's	Cleveland, OH	P	P	P	P	P	P	P	P	N	P	P	P	P	P	P
University of Michigan Health C.S. Mott Children's Hospital	Ann Arbor, MI	N	N	N	N	N	N	N	N	N	N	N	N	N	N	N
Nemours Children's Hospital-Delaware	Wilmington, DE	N	N	N	N	N	N	N	N	N	N	N	N	N	N	N
Nicklaus Children's Hospital	Miami, FL	N	N	N	N	N	N	N	N	N	N	N	N	N	N	N
Riley Hospital for Children at IU Health	Indianapolis, IN	P	P	P	P	P	P	P	P	P	P	P	P	P	P	P
The Bristol-Myers Squibb Children's Hospital at Robert Wood Johnson University Hospital	New Brunswick, NJ	N	N	N	N	N	N	N	N	N	N	N	N	N	N	N
University of Virginia Children's Hospital	Charlottesville, VA	P	P	N	P	P	P	N	N	P	P	N	N	P	P	N
MUSC Shawn Jenkins Children's Hospital	Charleston, SC	N	N	N	N	N	N	N	N	N	N	N	N	N	N	N
Seattle Children's	Seattle, WA	P	P	P	P	P	P	P	P	P	P	P	P	P	P	P
UCLA Mattel Children's Hospital	Los Angeles, CA	N	N	N	N	N	N	N	N	N	N	N	N	N	N	N
CHOC Children's	Orange County, CA	P	P	P	P	P	P	N	N	N	P	P	N	N	N	P
Cook Children's Medical Center	Fort Worth, TX	P	P	P	P	P	P	P	P	P	P	P	P	P	P	P
UCSF Benioff Children's Hospitals	San Francisco, CA	N	N	N	N	N	N	N	N	N	N	N	N	N	N	N
Kentucky Children's Hospital	Lexington, KY	P	P	N	N	P	P	N	N	N	P	P	N	P	P	N
OHSU Doernbecher Children's Hospital	Portland, OR	C	C	C	C	C	C	C	C	C	C	C	C	C	C	C
Norton Children's Hospital	Louisville, KY	N	N	N	N	N	N	N	N	N	N	N	N	N	N	N
Arkansas Children's Hospital	Little Rock, AR	P	P	P	N	P	P	N	N	P	P	N	N	N	N	P
Orlando Health Arnold Palmer Hospital for Children	Orlando, FL	N	N	N	N	N	N	N	N	N	N	N	N	N	N	N
Johns Hopkins All Children’s Hospital	St. Petersburg, FL	N	N	N	N	N	N	N	N	N	N	N	N	N	N	N
Ochsner Hospital for Children	New Orleans, LA	N	N	N	P	N	N	N	N	N	P	N	N	N	N	N
Joe DiMaggio Children's Hospital	Hollywood, FL	N	N	N	N	N	N	N	N	N	N	N	N	N	N	N
Cincinnati Children's Hospital Medical Center	Cincinnati, OH	P	P	P	P	P	P	P	P	P	P	P	P	P	P	P
Children's Medical Center Dallas-Scottish Rite for Children	Dallas, TX	C	C	C	C	C	C	C	C	C	C	C	C	C	C	C
Boston Children's Hospital	Boston, MA	P	N	N	P	P	P	N	N	N	N	P	P	N	P	N
Rady Children's Hospital-San Diego	San Diego, CA	N	N	N	N	N	N	N	N	N	N	P	N	N	N	N
Cohen Children's Medical Center	New Hyde Park, NY	P	P	N	N	P	P	N	N	N	N	P	N	P	P	P

Of the five requirements for the machine-readable CMS document, payer-specific negotiated charges, minimum negotiated charge, and maximum negotiated charge were the three CMS-required datapoints most often available to patients, with an average of 30% of hospitals listing them across all 15 CPT codes (Table 3). Meanwhile, an average of 18% of hospitals had information on discounted cash prices listed on their CMS document.

**Table 3:** Number of hospitals compliant per procedure and charge type

CPT code	Gross charge	Discounted cash price	Payer-specific negotiated charges	Minimum negotiated charge	Maximum negotiated charge
CPT 24538	12	8	15	14	15
CPT 24545	11	8	13	13	13
CPT 27506	9	7	11	10	10
CPT 27502	11	6	13	13	13
CPT 29888	11	7	14	14	14
CPT 29880	11	8	14	14	14
CPT 22802	7	7	9	9	9
CPT 22843	7	5	8	8	8
CPT 25574	8	7	10	10	10
CPT 25565	14	8	14	14	14
CPT 23462	9	7	13	13	13
CPT 29860	6	7	9	9	9
CPT 29914	9	8	12	12	12
CPT 29916	9	8	12	12	12
CPT 27176	8	7	11	11	11

Patient-friendly pricing tools were available on 46 (92%) hospital websites (Table 4). Additionally, all hospitals only provided price transparency information in English. All hospitals provided a phone number to call for specific pricing information. Forty (80%) hospitals provided a downloadable CMS chargemaster file. Of those downloaded, 16 (40%) were unusable due to issues, such as poor text readability, unlabeled pricing, or file-sizes exceeding 1GB, which led to computer freezes. The 24 (60%) remaining hospitals provided downloadable and usable files. Of the files that were available and usable, six (25%) did not have search capabilities (CPT, Diagnosis-Related Group [DRG], or keywords), and 10 (42%) only had keyword search capabilities.

**Table 4:** Compliance with CMS mandate for price transparency CMS, Centers for Medicare and Medicaid Services; DRG, Diagnosis-Related Group; CPT, Current Procedural Terminology

Variable	Percentage (number of hospitals)
Pricing tool	92(46)
CMS document	80(40)
Personal information required	14(7)
Description of procedure	30(15)
Phone number	100(50)
Language	
English	100(50)
English and Spanish	0(0)
English and other	0(0)
Time from initial query	
<5 min	60(30)
5-15 min	24(12)
>15 min	16(8)
Procedure search function	
Nothing	32(16)
CPT	4(2)
DRG	0(0)
Keywords	10(5)
Combination (keywords, DRG, and CPT)	54(27)

How do prices compare among the top 50 U.S. children’s orthopedic hospitals?

**Figure 1:**
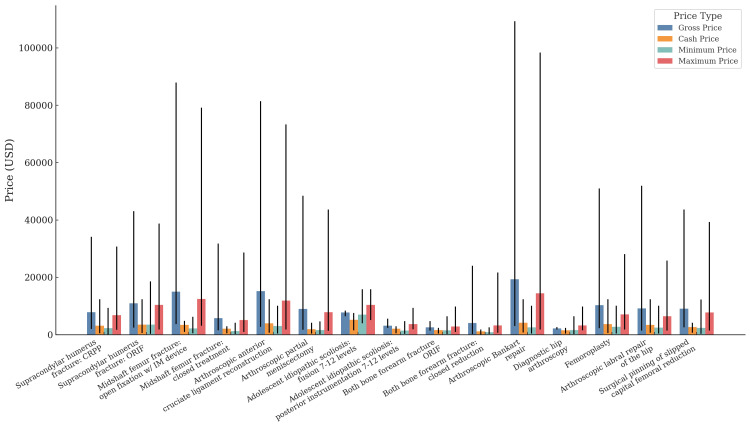
Price variability across common pediatric orthopedic procedures by price type Each bar represents the mean price for the respective category, with the error bars indicating the range from the minimum to the maximum price. Gross price is the total amount billed by the hospital for the procedure before any discounts or adjustments. Cash price is the charge for individuals paying cash without insurance. Minimum price is the lowest payer-negotiated price reported for the procedure across all insurers. Maximum price is the highest payer-negotiated price reported for the procedure across all insurers. Exact data used for this figure can be found in Table 5 in the Appendix.

The payer-negotiated charges were the most available datapoint, with 12 (24%) hospitals listing this data. The highest average maximum negotiated charge among the CPT codes of interest was CPT 23462 (arthroscopic Bankart repair), with a charge of $14,478 (Figure 1). The lowest average maximum negotiated charge was CPT 25574 (both bone forearm fracture ORIF), with a charge of $2,880 (Table 5, Appendix). There was substantial variability in maximum negotiated charges between the different hospitals. The widest range for the maximum negotiated charge was CPT 23462 (arthroscopic Bankart repair), ranging from $1,854 at one hospital to $98,388 at another. 

The highest average minimum negotiated charge among the CPT codes of interest was CPT 22802 (AIS: fusion 7-12 levels), with a charge of $7,040. The lowest average minimum negotiated charge was CPT 25565 (both bone forearm fracture closed reduction), with a charge of $830. The most varied, CPT 24545 (supracondylar humerus fracture: ORIF), ranged from $335 to $18,595 between hospitals (Figure 1).

The gross charge was the second most published datapoint, with 10 (20%) hospitals listing this information on their downloadable, machine-readable CMS document. CPT 23462 (arthroscopic Bankart repair) had the highest average gross charge of $19,368, and CPT 29860 (diagnostic hip arthroscopy) had the lowest average gross charge of $2,220. The widest range for gross charge was CPT 23462 (arthroscopic Bankart repair), ranging from $3,012 to $109,320. The narrowest range for the gross charge was CPT 29860 (diagnostic hip arthroscopy), ranging from $1,785 to $2,656 (Figure 1). 

Lastly, seven (14%) hospitals listed the cash price for each procedure. CPT 22802 (AIS: fusion 7-12 levels) had the highest average cost of $5,191, and CPT 25574 (both bone forearm fracture: ORIF) had the lowest average cost of $1,114. CPT 23462 (arthroscopic Bankart repair) had the most variability between cash prices, ranging from $766 to $12,403 (Figure 1).

## Discussion

Despite the 2019 Hospital Price Transparency legislation, recent literature demonstrates noncompliance with the mandates [2,4,8-12]. This study supports recent literature and shows that only five (10%) of the top 50 pediatric orthopedic hospitals provide adequate information to assess the price of common pediatric orthopedic procedures. While many hospitals had the required downloadable CMS required file, 16/40 of the files were deemed unusable. Of the 24 files providing some usability, 16 were severely limited by inability to search for a procedure, or requirement to search for a generic term that does not apply to the procedure of interest. Of the three mandated types of information, seven (14%) hospitals listed cash prices, 10 (20%) hospitals listed gross charges, and 12 (24%) hospitals listed payer-negotiated charges. Forty-six (92%) hospitals had some form of user-friendly pricing tools, but the ability to obtain a single price for a specific procedure was extremely limited. Furthermore, no hospitals offered pricing tools in any language other than English. Additionally, the cash price was the least commonly reported, further complicating the process of obtaining the price of a common pediatric orthopedic procedure for uninsured patients and/or those attempting to pay out of pocket.

This study seems to agree with other contemporary studies assessing price transparency after implementation of the CMS mandate. Most similarly, Ayoub and Balakrishnan conducted a study to evaluate pediatric hospital compliance with the CMS mandate, reporting 27% of the pediatric hospitals were compliant in pricing information for pediatric tonsillectomy [17]. Although this study reports compliance rates that are lower than those found in other manuscripts, the current study analyzed 15 different procedures, and it required pricing information for all procedures in order for a hospital to be deemed fully compliant. Furthermore, this study conducted individual assessments of the six mandates required by the CMS price transparency legislation, enabling the identification of precise areas where hospitals can enhance their price transparency practices and inform future enforcement strategies.

This study also shows a substantial variation in pricing for the 15 procedures of interest. For example, arthroscopic Bankart repair (23462) had the widest range for maximum negotiated charge, with a range from $1,854 to $98,388. For the same procedure, the cash price listed varied from $766 to $12,403. While minimum negotiated charges varied to a smaller extent, the dramatic differences seen among some negotiated rates are concerning, nonetheless. In general, the variability in pricing between hospitals and between cash prices, gross charges, and negotiated charges underscores the complex landscape of healthcare pricing [18]. The same procedure can result in vastly different costs depending on the institution. Not only is there a wide variability in terms of price for the same procedure, but there is also a lack of clear, easily accessible pricing information. This combination may deter patients from seeking necessary care or lead to excessive financial burdens, especially those that are uninsured or on high-deductible plans [19,20].

Limitations

There are some limitations to this study. First, this study only included the top pediatric orthopedic hospitals, which may not be the best representation for all children’s hospitals. Additionally, hospitals provide different quoted prices and charges based on the patient's insurance status or provider. While the CMS mandate requires hospitals to provide payer-specific charges, our study found that these specific price estimates were not available for most of the hospitals. Additionally, the price transparency legislation does not require estimates for professional fees for surgeons and anesthesiologists, so we were not able to evaluate these charges for the procedures. Furthermore, many of the pediatric hospitals evaluated in the study are affiliated with adult hospitals. These hospitals often merged the pediatric pricing data with the adult data, making it difficult to differentiate pediatric pricing from that of adult care.

## Conclusions

The current state of price transparency among top pediatric orthopedic hospitals falls significantly short of the expectations set by the CMS mandate. Compliance remains low, and even when hospitals attempt to meet requirements, significant barriers to accessibility limit the utility of the available pricing information. The observed inconsistencies in how hospitals report pricing further hinder patients’ ability to make informed financial decisions about their care. These findings highlight the need for a reassessment of current enforcement strategies and the practicality of existing regulations. Without meaningful improvements in transparency and usability, the intended benefits of price disclosure, such as empowering patients, reducing financial strain, and fostering competition, will remain largely unrealized. Future efforts should prioritize not only compliance but also the development of clear, standardized, and patient-friendly methods for accessing healthcare pricing information.

## Appendices

**Table 5:** Overview of pricing data for each procedure CPT, Current Procedural Terminology; CRPP, closed reduction percutaneous pinning; IM, intramedullary Table Credits: Nicholas Belt and Austin Lee

CPT code (procedure)	Price type	Min	Max	Mean
CPT 24538 (supracondylar humerus fracture: CRPP)	Gross price	2037	34,168	7856
	Cash price	504	12,403.01	3154
	Minimum price	0*	9347	2267
	Maximum price	1731	30,751	6766
CPT 24545 (supracondylar humerus fracture: ORIF)	Gross price	2508	43,069	10,978
	Cash price	584	12,403	3529
	Minimum price	336	18,595	3533
	Maximum price	1855	38,762	10,357
CPT 27506 (midshaft femur fracture: open fixation with IM device)	Gross price	3719	87,933	15,021
	Cash price	949	4797	3410
	Minimum price	498	6265	2218
	Maximum price	3161	79,139	12,488
CPT 27502 (midshaft femur fracture: closed treatment)	Gross price	1552	31,808	5813
	Cash price	514	2979	2050
	Minimum price	244	4189	1286
	Maximum price	947	28,627	5109
CPT 29888 (arthroscopic anterior crucial ligament reconstruction)	Gross price	2778	81,429	15,211
	Cash price	708	12,403	3953
	Minimum price	372	10,142	3031
	Maximum price	1855	73,286	11,903
CPT 29880 (arthroscopic partial meniscectomy)	Gross price	1724	48,486	9020
	Cash price	402	4134	1947
	Minimum price	252	4668	1614
	Maximum price	1360	43,637	7871
CPT 22802 (adolescent idiopathic scoliosis: fusion 7-12 levels)	Gross price	6461	8371	7734
	Cash price	1497	7534	5191
	Minimum price	3934	15,868	7040
	Maximum price	5128	15,868	10,350
CPT 22843 (adolescent idiopathic scoliosis: posterior instrumentation 7-12 levels)	Gross price	2418	5600	3169
	Cash price	526	2925	2103
	Minimum price	324	4764	1430
	Maximum price	1932	9343	3705
CPT 25574 (both bone forearm fracture: ORIF)	Gross price	1459	4761	2564
	Cash price	426	2411	1645
	Minimum price	239	6397	1511
	Maximum price	966	9849	2880
CPT 25565 (both bone forearm fracture: closed reduction)	Gross price	187	24,092	4074
	Cash price	140	1782	1114
	Minimum price	182	2543	830
	Maximum price	714	21,682	3262
CPT 23462 (arthroscopic Bankart repair)	Gross price	3012	109,320	19,368
	Cash price	766	12,403	4138
	Minimum price	403	10,142	2594
	Maximum price	1855	98,388	14,479
CPT 29860 (diagnostic hip arthroscopy)	Gross price	1785	2656	2220
	Cash price	375	2391	1552
	Minimum price	239	6397	1666
	Maximum price	1517	9849	3220
CPT 29914 (femoroplasty)	Gross price	2315	50,958	10,295
	Cash price	635	12,403	3724
	Minimum price	416	10,142	2737
	Maximum price	1774	28,117	7053
CPT 29916 (arthroscopic labral repair of the hip)	Gross price	1431	51,987	9134
	Cash price	647	12,403	3373
	Minimum price	424	10,142	2490
	Maximum price	1402	25,883	6477
CPT 27176 (surgical pinning of slipped capital femoral reduction)	Gross price	2546	43,658	9081
	Cash price	638	4134	2633
	Minimum price	341	12,315	2347
	Maximum price	1479	39,292	7773

**Table 6:** Comprehensive characteristics for hospitals included in this study Table Credits: Nicholas Belt and Austin Lee

Hospital name	U.S. census region	Public vs. private	Bed size	Urban vs. rural	Teaching hosptial	Profit or non-profit
Texas Children's Hospital	South	Private	973	Urban	Yes	Non-profit
Johns Hopkins Children's Center	South	Private	209	Urban	Yes	Non-profit
Children's Healthcare of Atlanta	South	Private	446	Urban	Yes	Non-profit
Nationwide Children's Hospital	Midwest	Private	674	Urban	Yes	Non-profit
UH Rainbow Babies and Children's Hospital	Midwest	Private	244	Urban	Yes	Non-profit
St. Louis Children's Hospital-Washington University	Midwest	Private	402	Urban	Yes	Non-profit
UPMC Children's Hospital of Pittsburgh-Shriners Hospitals for Children Erie	Northeast	Private	313	Urban	Yes	Non-profit
Children’s Hospital Colorado	West	Private	284	Urban	Yes	Non-profit
Mayo Clinic Children's Center	Midwest	Private	148	Urban	Yes	Non-profit
Lerner Children's Pavilion-Hospital for Special Surgery	Northeast	Private	Unknown	Urban	Yes	Non-profit
Children's Mercy Kansas City	Midwest	Private	367	Urban	Yes	Non-profit
Levine Children's Hospital	South	Public	247	Urban	Yes	Non-profit
UC Davis Children's Hospital/Shriners Children's Northern California	West	Private	158	Urban	Yes	Non-profit
North Carolina Children's Hospital at UNC	South	Public	150	Urban	Yes	Non-profit
Monroe Carell Jr. Children’s Hospital at Vanderbilt	South	Private	343	Urban	Yes	Non-profit
Duke Children's Hospital and Health Center	South	Public	190	Urban	Yes	Non-profit
Lucile Packard Children's Hospital Stanford	West	Private	361	Urban	Yes	Non-profit
Valley Children's Healthcare and Hospital	West	Private	358	Urban	Yes	Non-profit
Dayton Children's Hospital	Midwest	Private	181	Urban	Yes	Non-profit
NewYork-Presbyterian Children's Hospital-Columbia and Cornell	Northeast	Private	299	Urban	Yes	Non-profit
University of Iowa Stead Family Children's Hospital	Midwest	Public	190	Urban	Yes	Non-profit
Cleveland Clinic Children's	Midwest	Private	389	Urban	Yes	Non-profit
University of Michigan Health C.S. Mott Children's Hospital	Midwest	public	348	Urban	Yes	Non-profit
Nemours Children's Hospital, Delaware	South	Private	130	Urban	Yes	Non-proft
Nicklaus Children's Hospital	South	Private	307	Urban	Yes	Non-profit
Riley Hospital for Children at IU Health	Midwest	Public	456	Urban	Yes	Non-profit
The Bristol-Myers Squibb Children's Hospital at Robert Wood Johnson University Hospital	Northeast	Public	105	Urban	Yes	non-profit
University of Virginia Children's Hospital	South	Public	112	Urban	Yes	Non-profit
MUSC Shawn Jenkins Children's Hospital	South	Public	250	Urban	Yes	Non-profit
Seattle Children's Hospital	West	Private	407	Urban	Yes	Non-profit
UCLA Mattel Children's	West	Private	156	Urban	Yes	Non-profit
CHOC Children's	West	Private	334	Urban	Yes	Non-profit
Cook Children's Medical Center	South	Private	443	Urban	Yes	Non-profit
UCSF Benioff Children's Hospitals	West	Public	183	Urban	Yes	Non-profit
Kentucky Children's Hospital	South	Private	205	Urban	Yes	Non-profit
OHSU Doernbecher Children's Hospital	West	Public	151	Urban	Yes	Non-profit
Norton Children's Hospital	South	Private	300	Urban	Yes	Non-profit
Arkansas Children's Hospital	South	Private	336	Urban	Yes	Non-profit
Orlando Health Arnold Palmer Hospital for Children	South	Private	158	Urban	Yes	Non-profit
Johns Hopkins All Children’s Hospital	South	Private	259	Urban	Yes	Non-profit
Ochsner Hospital for Children	South	Private	125	Urban	Yes	Non-profit
Joe DiMaggio Children's Hospital at Memorial	South	Public	216	Urban	Yes	Non-profit
Cincinnati Children's Hospital Medical Center	Midwest	Private	670	Urban	Yes	Non-profit
Children's Medical Center Dallas-Scottish Rite for Children	South	Private	100	Urban	Yes	Non-profit
Boston Children's Hospital	Northeast	Private	477	Urban	Yes	Non-profit
Rady Children's Hospital-San Diego	West	Private	511	Urban	Yes	Non-profit
Cohen Children's Medical Center	Northeast	Private	202	Urban	Yes	Non-profit
Children’s Hospital of Philadelphia	Northeast	Private	594	Urban	Yes	Non-profit
Children’s Hospital Los Angeles	West	Private	495	Urban	Yes	Non-profit
Children's National Hospital	South	Private	303	Urban	Yes	Non-profit
